# A pilot investigation using global positioning systems into the outdoor activity of people with severe traumatic brain injury

**DOI:** 10.1186/1743-0003-11-37

**Published:** 2014-03-19

**Authors:** Ross A Clark, Natasha Weragoda, Kade Paterson, Stacey Telianidis, Gavin Williams

**Affiliations:** 1 School of Exercise Science, Australian Catholic University, 3065 Melbourne, Australia; 2 Epworth HealthCare, Richmond, Australia; 3 School of Physiotherapy, University of Melbourne, Melbourne, Australia

**Keywords:** Physical activity, Activity monitor, GPS, Community mobility, HiMAT

## Abstract

**Background:**

Little is known about the post-discharge outdoor activities of people who have incurred severe traumatic brain injury (TBI). This study used a body-worn global positioning system (GPS) device to determine the outdoor activity per day performed by this population. Additionally, this study examined the association that mobility, time since injury and injury severity had with levels of outdoor physical activity.

**Findings:**

Seventeen people with TBI and 15 control subjects wore a GPS device for between 3–7 days to monitor their outdoor activity. Based on the individual’s location and speed of movement the outdoor physical activity in minutes per day was derived. Assessments of duration of outdoor activity between groups, and the relationship that duration of outdoor activity had with results on the high-level mobility assessment tool, length of post-traumatic amnesia, and time since injury were performed. No significant (p = 0.153, effect size = 0.26) difference in time spent in outdoor physical activity was observed between the TBI (median[IQR] = 19[3–43]mins) and control (median[IQR] = 50[18–65]mins) group. Interestingly, 35% of TBI subjects performed <10 mins of outdoor activity per day compared to 13% of the control group. The TBI group also recorded three of the four highest values for outdoor physical activity. Higher levels of mobility were associated with more outdoor activity (Spearman’s rho = 0.443, p = 0.038). No other significant associations were observed.

**Conclusions:**

While preliminary, our results indicate that a sub-group of people with TBI exists who restrict their outdoor activities. GPS has potential as an activity tracking tool, with implications for rehabilitation and exercise prescription.

## Introduction

Following severe TBI people perform less physical activity [1], and due to functional mobility limitations their opportunities to perform outdoor activity may be reduced. Outdoor activity has numerous established benefits, ranging from obtaining Vitamin D [2] through to maintaining cardiovascular fitness and reducing depressive symptoms [3]. In people living with a severe traumatic brain injury (TBI), participating in outdoor activity is also an important step towards community integration [4]. Self-report assessment of outdoor activity is simple to obtain and valid in many populations [5,6], however problems associated with memory and recall following severe TBI limit this method of data collection [7]. Measures such as self-selected walking speed are often used to predict functional gait performance and capacity to walk outdoors [8], however largely due to the difficulties associated with accurate data collection little quantitative evidence of the actual outdoor activity levels of this population exists.

Recent advances in “big brother” technology such as lightweight global positioning systems (GPS) allow for precise tracking of outdoor movement. Wearable GPS systems have also been shown to provide valid information, and are feasible to use in people living with stroke [9]. Despite evidence that many people with a clinical condition report a reduction in their outdoor activity and a more sedentary indoor lifestyle [10], there is limited research using GPS in these populations [11-13], and none in people following severe TBI.

The aim of this study was to compare the outdoor activity of a TBI population with a matched healthy control group by using GPS tracking, and to examine whether functional mobility levels, injury severity and time since injury were associated with greater outdoor activity in this group. We hypothesised that 1) the TBI cohort would perform less physical activity outdoors, with a large number of participants performing very little outdoor physical activity, and 2) that improved physical function, lesser injury severity and a longer time since injury would be associated with increased outdoor activity.

## Methods

A convenience sample of 17 independently ambulant people with severe TBI attending an outpatient brain injury physiotherapy department for their mobility restrictions, and 15 control subjects recruited by advertising and word-of-mouth matched for age and pre-injury employment status [14] provided informed consent to volunteer for this study. All study protocols were approved by Epworth Hospital (HREC No: 43609) and the Australian Catholic University Human Research Ethics Committee (No. V201072).

Participants were asked to wear a Kinetamap GPS Unit (Sparkfun Electronics, U.S.A.) sampling GPS data at 1 Hz for 7 consecutive days. This system uses a 20 Channel EM-408 SiRF III Receiver, with a cold, warm and hot start speed of 42, 38 and 8 seconds respectively. Given that the unit was kept on for the duration of the day, and that satellites are often detected whilst indoors, once the initial GPS signal was detected the typical lag time for outdoor detection was either 8 seconds (hot start time if no indoor satellite detection was available) or near instantaneous (if transitioning from an indoor environment with a satellite fix). To prolong battery life the unit was modified to use a larger 2000mAh lithium polymer battery, providing continuous usage time of ≈ 20 hrs. Overnight recharging was performed via a mains-powered recharger.

A minimum data collection threshold of wearing the unit >8 hrs/day for ≥3 days in the one week period was set, as it is common for people to forget to wear activity monitors during longitudinal studies and this timeframe meets the recommended minimum criteria for pedometer-based activity monitoring [15]. The monitor, which is roughly the size of a mobile phone, was worn in a money belt around the waist from the time the participant woke-up in the morning until they went to bed and was left on constantly.

**Figure 1:**
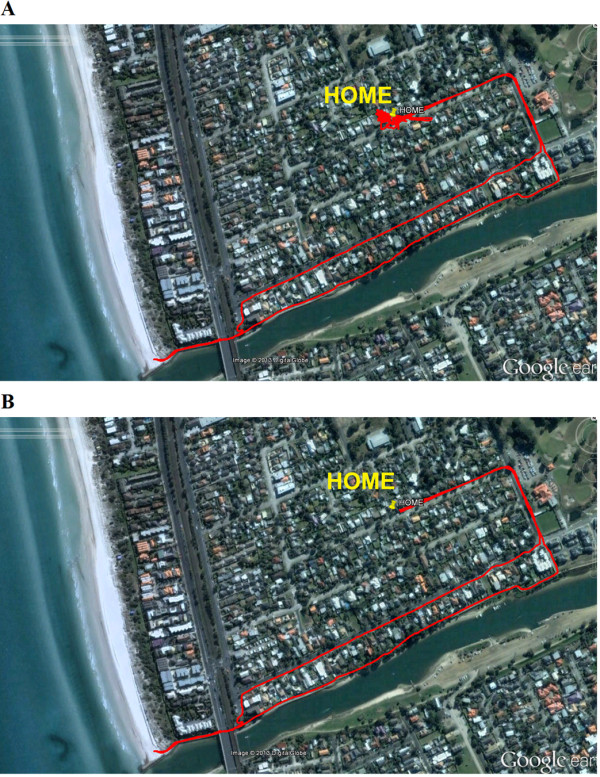
**Example of the raw and analysed GPS data for a participant uploaded into Google Earth.** For this trial analysis consisted of removing the indoor data, as evidenced in **(A)** by the noisy oscillations - indicative of poor indoor GPS signal - about the HOME position marked by the yellow pin. These images were uploaded into Google Earth by a custom software program which was used to trim the files. The velocity and location of movement was assessed in this program using the GPS data, and subjective decisions about the mode of activity (physically active, passive transport) were made based on these data. In this example the velocity of movement never exceeded 5 km/hr, and the location was predominantly on a walking track identified using Google Earth, and therefore it was deemed to be outdoor physical activity **(B)**.

At completion of the one week period these data were uploaded by a blinded assessor into a custom LabVIEW program (National Instruments, U.S.A.) synchronised with Google Earth, and the custom program was used to distinguish between indoor time, physical activity performed outdoors and non-activity related transport by evaluating the location, velocity of movement and number of satellites with fixes. Specifically, all GPS data was loaded into this program, and segments which were identified as being indoors were removed from the signal. The remaining data constituted being outdoors, and subjective assessment of location and velocity of movement was used to further extract data which was likely to be non-physical activity (such as driving on a highway) [16]. These passive transport data were removed to ensure that our results represented only physical activity performed outdoors. An example of how the program was used to identify outdoor activity is provided in Figure 1.

Physical function in the TBI group was assessed using a measure of functional mobility specifically designed for this population, the high level mobility assessment tool (HiMAT). This tool assesses performance on movements ranging from walking to jumping, and the results are reliable [17]. Severity of TBI was quantified by length of post-traumatic amnesia (PTA).

Descriptive statistics for age, post-traumatic amnesia (PTA) duration, time post injury and HiMAT score are reported as median[interquartile range]. Due to the small sample size in each group, statistical analysis consisted of nonparametric one-tailed Mann Whitney U, skewness and kurtosis assessments to compare results between the two groups, and Spearman’s correlation analysis within the TBI group to examine associations [18]. Analysis was performed in SPSS V20, with the significance threshold set at p < 0.05.

## Results

Descriptive statistics for our TBI group were age = 28[24–38]yrs, PTA duration = 34[16–79]days, time post injury = 437[292–1126]days, HiMAT score = 30[19.5-38.8], males = 11. Nine and two members of the TBI and control groups were not employed/students respectively.

**Figure 2:**
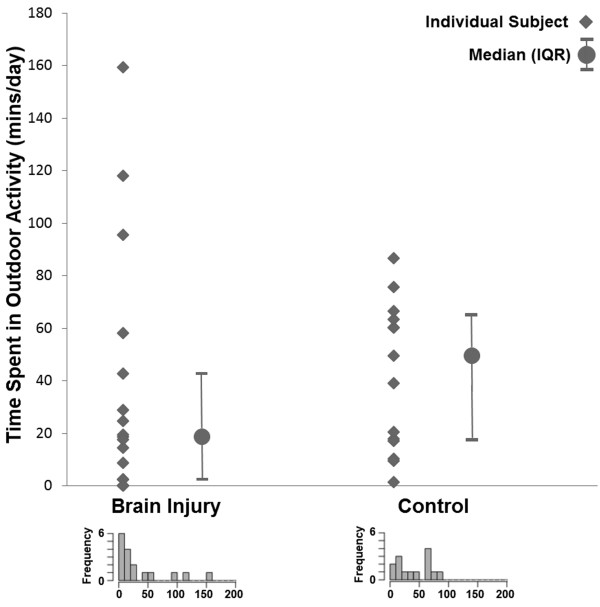
**Individual subject, group median and group inter-quartile range results for time spent in outdoor activity per day.** Distributions are plotted in the histograms at the bottom of the graph in 10 minute blocks, and were created using an online histogram plotting tool (http://www.wessa.net/rwasp_histogram.wasp). Note: outlying data for one control subject (value = 441 mins/day) removed from the graph but included in the non-parametric statistical analysis.

Both groups wore the device for 5 ± 2 days. Per day the TBI group wore the device for 9.0 ± 1.9 hrs, the control group for 9.0 ± 4.8 hrs. The individual subject and median with inter-quartile range results for the time spent in outdoor physical activity per day are provided in Figure 2. No significant (p = 0.153, Z = 1.45, effect size = 0.26) difference in time spent in outdoor physical activity was observed between the TBI (median[IQR] = 19[3–43]mins) and control (median[IQR] = 50[18–65]mins) group. Interestingly, the distribution of the results was markedly different between groups, as evidenced by the distribution of individual subject values in Figure 2 and the skewness (skewness[SE]: TBI = 1.70[0.55], control = 3.45[0.58]) and kurtosis (kurtosis[SE]: TBI = 2.21[1.06], control = 12.69[1.12]) values for each group. The distribution for the TBI group was noticeably peaked in the low levels of activity, with 35% and 71% of TBI subjects performing less than 10 mins and 30 mins of outdoor activity per day respectively. This contrasts poorly with the control group, in which 13% and 40% of participants were under these respective thresholds. Statistically this was offset by the TBI group also having a number of participants performing a high amount of outdoor physical activity, recording three of the four highest scores on this measure.

A significant, moderate strength correlation (rho = 0.443, p = 0.038) was observed between the HiMAT and time spent in outdoor activity, indicating that people with higher levels of mobility perform more outdoor activity. A trend was observed for an association between time spent in outdoor activity and PTA (rho = -0.384, p = 0.064), with increased duration of amnesia associated with a reduction in outdoor activity levels. A weak, non-significant association was observed between time since injury and time spent in outdoor activity (rho = -0.359, p = 0.157).

## Discussion

This is the first study to quantitatively assess outdoor physical activity levels in a TBI population using GPS. The TBI participants performed less outdoor physical activity than the control subjects; however this did not reach significance and therefore does not fully support our first hypothesis. There was a distinct difference in the pattern of distribution of the results, with the TBI group having a very high proportion of people performing little outdoor activity and a small proportion performing a large amount relative to the control group. This is likely influenced by the ability of the person with TBI to perform physical activity, as indicated by the significant association between functional mobility and time spent in outdoor activity which supports our second hypothesis.

The clustering in the low levels of activity for the TBI group is indicative of a cohort who, despite an increased amount of available “leisure” time due to unemployment (53% not working in any paid capacity), has restricted their outdoor activities. This result supports the previous subjective findings of Eriksson et al. [10], who reported a 24% reduction in outdoor life by their TBI population. Given the multi-dimensional and cognitive demands for outdoor walking, these findings reinforce that a prescribed outdoor rehabilitation program may need to be considered for this sub-group. In this respect the GPS system could form an invaluable tool for the rehabilitation practitioner, with the ability to prescribe and monitor outdoor activity using specific courses located near the person’s home if they are not performing an adequate amount of outdoor activity. For example, an accessible recreational park could be identified near the person’s home using Google Earth, and a walking track devised that provides sufficient stimulus to the participant based on their level of mobility. However, the ethical implications of tracking people’s activity must be considered.

This study had limitations, including our decision to focus on only outdoor activity. Therefore our study does not take into account indoor exercise, which may constitute a higher proportion of overall physical activity in a clinical population who have restricted mobility. We also focused on physical activity capacity, as measured using the HiMAT, and injury related factors. Consequently, no examinations of other important intrinsic or extrinsic factors such as cognitive status and environmental barriers to physical activity which may have influenced physical activity levels were performed. Additionally, we did not control for or record the days that the participant did not wear the device, and therefore our results may be biased if one group wore the device more on weekends [19]. We also used informed, but subjective, assessment to determine incidences of outdoor physical activity based on location, velocity of movement and the number of satellites fixes. This combination is commonly used in GPS studies of human movement [16]. While this is likely to discriminate between activities such as walking and driving, if participants performed movements such as riding a bicycle at high speeds we may have incorrectly excluded these data. There is also the potential for drop-out when using GPS near large buildings or in dense urban areas [16]. Although we used a relatively high quality GPS receiver, we cannot be certain that some outdoor activity was not incorrectly labelled as occurring indoors. Finally, we had a modest subject number and restricted data capture. This was due in large part to the device being used to assess activity, which at the time of purchase and software design (2010–2011) was a cutting edge activity monitoring tool. However, its size (28 × 63 × 94 mm) meant that it was unwieldy, and many potential research participants refused to wear it. Additionally, it required recharging overnight which may have resulted in missed days of data collection. These issues may be overcome using advances in low-power GPS tracking devices, with great potential for Smartphones to be programmed for monitoring and real-time feedback.

## Abbreviations

GPS: Global positioning system; TBI: Traumatic brain injury; PTA: Post-traumatic amnesia; HiMAT: High level mobility assessment tool; SE: Standard error.

## Competing interests

The authors declare that they have no competing interests.

## Authors’ contributions

RC created the data collection and analysis system, performed the statistical analysis and drafted the manuscript. NW organised subject recruitment and collected data and provided input into the drafting process. KP supervised the data collection and provided input into the drafting process. ST processed and analysed the data and provided input into the drafting process. GW was the senior researcher overseeing the project, assisted in subject recruitment and retention and provided input into the drafting process. All authors read and approved the final manuscript.

## References

[B1] Williams G Weragoda N Paterson K Clark R Cardiovascular fitness is unrelated to mobility limitations in ambulant people with traumatic brain injury J Head Trauma Rehabil 2013 28 E1 E7 23348405 10.1097/HTR.0b013e318279536d

[B2] McCurdy LE Winterbottom KE Mehta SS Roberts JR Using nature and outdoor activity to improve children’s health Curr Probl Pediatr Adolesc Child Care 2010 40 102 117 10.1016/j.cppeds.2010.02.003 20381783

[B3] Pretty J Peacock J Hine R Sellens M South N Griffin M Green exercise in the UK countryside: Effects on health and psychological well-being, and implications for policy and planning J Environ Plan Manage 2007 50 211 231 10.1080/09640560601156466

[B4] McColl MA Carlson P Johnston J Minnes P Shue K Davies D Karlovits T The definition of community integration: perspectives of people with brain injuries Brain Inj 1998 12 15 30 10.1080/026990598122827 9483334

[B5] Washburn RA McAuley E Katula J Mihalko SL Boileau RA The physical activity scale for the elderly (PASE): evidence for validity J Clin Epidemiol 1999 52 643 651 10.1016/S0895-4356(99)00049-9 10391658

[B6] Schreuer N Sachs D Rosenblum S Participation in leisure activities: Differences between children with and without physical disabilities Res Dev Disabil 2013 35 223 233 24176261 10.1016/j.ridd.2013.10.001

[B7] Vakil E The effect of moderate to severe traumatic brain injury (TBI) on different aspects of memory: A selective review J Clin Exp Neuropsychol 2005 27 977 1021 10.1080/13803390490919245 16207622

[B8] Perry J Garrett M Gronley JK Mulroy SJ Classification of walking handicap in the stroke population Stroke 1995 26 982 989 10.1161/01.STR.26.6.982 7762050

[B9] McCluskey A Ada L Dean CM Vargas J Feasibility and validity of a wearable GPS device for measuring outings after stroke ISRN Rehabil 2012 2012

[B10] Eriksson G Tham K Borg J Occupational gaps in everyday life 1–4 years after acquired brain injury J Rehabil Med 2006 38 159 165 10.1080/16501970500415322 16702082

[B11] Neven A Janssens D Alders G Wets G Wijmeersch BV Feys P Documenting outdoor activity and travel behaviour in persons with neurological conditions using travel diaries and GPS tracking technology: A pilot study in multiple sclerosis Disabil Rehabil 2013 35 1718 1725 10.3109/09638288.2012.751137 23343357

[B12] Shoval N Wahl HW Auslander G Isaacson M Oswald F Edry T Landau R Heinik J Use of the global positioning system to measure the out-of-home mobility of older adults with differing cognitive functioning Ageing Soc 2011 31 849 869 10.1017/S0144686X10001455

[B13] Miskelly F Electronic tracking of patients with dementia and wandering using mobile phone technology [1] Age Ageing 2005 34 497 499 10.1093/ageing/afi145 16107453

[B14] McLennan W Australian Standard Classification of Occupations 1997 Commonwealth of Australia: Australian Standard Classification of Occupations

[B15] Tudor-Locke C Burkett L Reis JP Ainsworth BE Macera CA Wilson DK How many days of pedometer monitoring predict weekly physical activity in adults? Prev Med 2005 40 293 298 10.1016/j.ypmed.2004.06.003 15533542

[B16] Kerr J Duncan S Schipperjin J Using global positioning systems in health research: A practical approach to data collection and processing Am J Prev Med 2011 41 532 540 10.1016/j.amepre.2011.07.017 22011426

[B17] Williams GP Greenwood KM Robertson VJ Goldie PA Morris ME High-level mobility assessment tool (HiMAT): Interrater reliability, retest reliability, and internal consistency Phys Ther 2006 86 395 400 16506875

[B18] Pett MA Nonparametric statistics in health care research: Statistics for small samples and unusual distributions 1997 London: Sage

[B19] Isaacs K McCurdy T Glen G Nysewander M Errickson A Forbes S Graham S McCurdy L Smith L Tulve N Vallero D Statistical properties of longitudinal time-activity data for use in human exposure modeling J Exposure Sci Environment Epidemiol 2013 23 328 336 10.1038/jes.2012.94 23047319

